# Genetic structure and landscape effects on gene flow in the Neotropical lizard *Norops brasiliensis* (Squamata: Dactyloidae)

**DOI:** 10.1038/s41437-024-00682-5

**Published:** 2024-04-04

**Authors:** Emanuel M. Fonseca, Nathaniel S. Pope, William E. Peterman, Fernanda P. Werneck, Guarino R. Colli, Bryan C. Carstens

**Affiliations:** 1 Department of Evolution, Ecology and Organismal Biology, The Ohio State University, Columbus, OH USA; 2 Institute of Ecology and Evolution, University of Oregon, Eugene, OR 97403 USA; 3 School of Environment and Natural Resources, The Ohio State University, Columbus, OH USA; 4 Coordenação de Biodiversidade, Programa de Coleções Científicas Biológicas, Instituto Nacional de Pesquisas da Amazônia (INPA), Manaus, Brazil; 5 Departamento de Zoologia, Universidade de Brasília, Brasília, Brazil

**Keywords:** Genetic variation, Evolutionary genetics

## Abstract

One key research goal of evolutionary biology is to understand the origin and maintenance of genetic variation. In the Cerrado, the South American savanna located primarily in the Central Brazilian Plateau, many hypotheses have been proposed to explain how landscape features (e.g., geographic distance, river barriers, topographic compartmentalization, and historical climatic fluctuations) have promoted genetic structure by mediating gene flow. Here, we asked whether these landscape features have influenced the genetic structure and differentiation in the lizard species *Norops brasiliensis* (Squamata: Dactyloidae). To achieve our goal, we used a genetic clustering analysis and estimate an effective migration surface to assess genetic structure in the focal species. Optimized isolation-by-resistance models and a simulation-based approach combined with machine learning (convolutional neural network; CNN) were then used to infer current and historical effects on population genetic structure through 12 unique landscape models. We recovered five geographically distributed populations that are separated by regions of lower-than-expected gene flow. The results of the CNN showed that geographic distance is the sole predictor of genetic variation in *N. brasiliensis*, and that slope, rivers, and historical climate had no discernible influence on gene flow. Our novel CNN approach was accurate (89.5%) in differentiating each landscape model. CNN and other machine learning approaches are still largely unexplored in landscape genetics studies, representing promising avenues for future research with increasingly accessible genomic datasets.

## Introduction

Disentangling the processes and mechanisms that influence genetic variation is critical to understanding biodiversity. Despite significant recent advances, the Northern Hemisphere remains the primary focal region for most phylogeographic and landscape genetic research (Beheregaray 2008; Storfer et al. 2010). Investigations on the origins of genetic, functional, and phylogenetic diversity in many taxonomic groups in the Southern Hemisphere are still wanting. This is unfortunate because the historical and ecological processes that operate in higher latitudes may differ from those in lower latitudes given the higher diversification rates, older ages, and more complex biotic interactions in the latter (Brown 2014; Jablonski et al. 2006; Rangel et al. 2018). New investigations offer the opportunity to test the generality of previous findings and may expand the view of the historical processes that have operated over time.

Landscape genetics is a discipline that seeks to understand how spatial and temporal variation in landscape features has shaped genetic variation by influencing biological processes such as dispersal and mating. One of the most well-documented patterns in population genetics is the decrease of genetic similarity among populations as the geographic distance between them increases (isolation by distance – IBD; Wright 1943). While IBD reflects spatially assortative mating due to limited dispersal in a homogeneous landscape, most natural landscapes are a mosaic of suitable habitats surrounded by an unsuitable habitat matrix that may constrain dispersal among local populations in a non-linear manner. For example, valleys can shape genetic differentiation in high-elevation species by constraining gene flow among mountain chains. Thus, landscape composition and configuration typically modulate the movement of individuals across space, leading to patterns of genetic differentiation that not only track the effect of geographic separation but also reflect the potential impact of habitat suitability on dispersal (isolation by resistance – IBR; McRae 2006). Therefore, the complex interaction between IBD and IBR can affect microevolutionary processes, such as gene flow, and influence the distribution of genetic variation at different temporal and spatial scales (Manel et al. 2003; Manel and Holderegger 2013).

The Cerrado, a world biodiversity hotspots (Myers et al. 2000), is a habitat located primarily in the Central Brazilian Plateau that covers about 22% of the Brazilian territory (Oliveira and Marquis 2002). It is composed of a complex landscape of older plateaus and younger depressions and dominated by sclerophyllous, fire-adapted vegetation, abundant grasses, and short, thick-barked, and twisting trees. Within the Cerrado, previous investigations have shown how rivers, environmental conditions, historical climate, fragmentation, and habitat loss explain genetic differentiation in disparate taxa (Telles et al. 2014; Vasconcellos et al. 2019; Fonseca et al. 2021). One of the main hypothesized biogeographical barriers is the topographic compartmentalization of the landscape into plateaus and valleys (Silva 1995; Werneck 2011). Plateaus are older features and are dominated by savanna-like vegetation, while valleys are much younger and characterized by more heterogeneous vegetation, including forests. The environmental transition between plateaus and valleys is steep; therefore, valleys presumably prevent gene flow between populations inhabiting plateaus and vice-versa. Another well-known effective barrier to gene flow is rivers—riverine barrier hypothesis (RBH; Wallace 1852). The RBH explain patterns of geographic distribution and genetic variation of several taxa worldwide (Gehring et al. 2012; Bartáková et al. 2015; Satler and Carstens 2016; Lanna et al. 2020). In addition to plateaus and rivers, areas characterized by climate-induced habitat instability likely limited gene flow between areas with greater habitat stability (Vitorino et al. 2018; Vasconcellos et al. 2019; Ledo et al. 2020). Hence, the combination of physical barriers and historical habitat instability may be expected to influence connectivity among populations, driving genetic differentiation through IBR. Lastly, considering the sheer area of the Cerrado (ca. 2,000,000 km^2^) and the limited dispersal capabilities of many taxa, IBD is also expected to be one of the most substantial factors affecting genetic variation in this region.

Here we investigate the role of landscape features on genetic structure and differentiation in an anole lizard species (*Norops brasiliensis*: Squamata: Dactyloidae) distributed throughout the Cerrado. To accomplish this objective, we use genetic clustering analysis, spatial analyses to estimate levels of gene flow across the landscape, and AMOVAs to assess genetic structure. We also calculate genetic summary statistics to describe overall genetic diversity in the focal species. Finally, we use optimized isolation-by-resistance models via maximum likelihood and a novel machine learning approach to test the effect of five landscape and environmental features on genetic differentiation: (i) geographic distance; (ii) geomorphological compartmentalization; (iii) rivers; (iv) vegetational shifts due to climatic oscillations, and (v) environmental niche suitability since the last glacial maximum.

## Material and methods

**Fig. 1:**
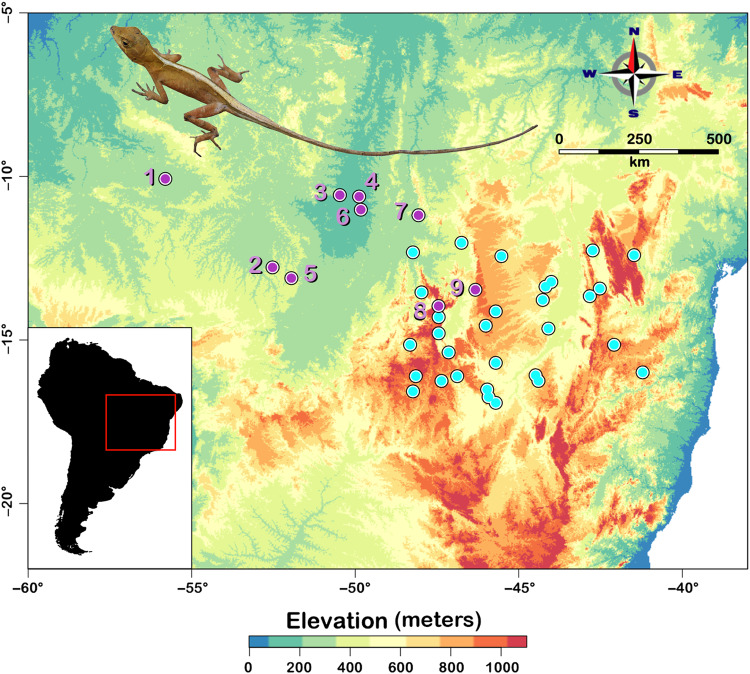
Map showing the geographic distribution of *Norops brasiliensis.* Localities with genetic data are shown in purple and museum records are shown in cyan.

*Norops brasiliensis* is a trunk-ground lizard species found in forested areas in the Cerrado, in enclaves of Cerrado within Amazonia, and in transitional areas between them (Fig. 1; Avila-Pires 1995).

### Sampling and data collection

We previously obtained 52 tissue samples from 9 localities (mean ± sd: 5.7 individuals per locality, range 1–18) of *N. brasiliensis* in the Cerrado and peripheral Cerrado enclaves in Amazonia as described by Fonseca et al. (2021). Sampling localities were drawn from across the range of the species but were limited by the availability of specimens. Genomic data were generated using a modified version of the Genotyping-by-Sequencing protocol described in Elshire et al. (2011). Briefly, this protocol sequences single nucleotide polymorphisms from throughout the genome that are adjacent to the Sbf1 restriction site. As described in Fonseca, Colli, et al. (2021), ipyrad v 0.9.52 (Eaton and Overcast 2020) was used to conduct all bioinformatic processing. We used the same configuration as described in Fonseca et al. (2021). First, we demultiplexed raw data using individual barcode adapters. Next, we filtered the data for adapters using the stricter option. We set the maximum low-quality base calls in the read to five and we only allowed reads longer than 35 bp. We clustered reads within each sample if their similarity was higher than 85%. We set maximum cluster depth within samples to 10,000 reads and used a minimum depth for statistical base calling of six reads. The Ohio Supercomputer Center provided the computational resources for processing all steps. Additionally, to supplement sample collection localities, we obtained 30 independent geographic distribution records from the literature and the Herpetological Collection, University of Brasília (CHUNB) used in the simulations (below).

### Genetic structure and genetic diversity

We reassessed the genetic structure in *N. brasiliensis* using the model-based clustering method sNMF implemented in the R package *LEA* (Frichot et al. 2014; Frichot and François 2015). sNMF is accurate likelihood algorithm that estimates genetic ancestry components for each sample using sparse non-negative matrix factorization and least-squares optimization. We performed fifteen independent runs, evaluating *K*-values ranging from 1 to 10. We selected the number of populations that minimized the cross-entropy criterion. Since clustering methods are likely to be sensitive to isolation by distance, we explored a range of clustering levels.

We calculated mean expected (H_e_) and observed (H_o_) heterozygosity within the SNP data based on the populations recovered by sNMF using the R package *hierstat* (Goudet 2005). We used an analysis of molecular variance (AMOVA) to assess the level of population structure among individual demes and the populations identified by sNMF using the R package *poppr* (Kamvar et al. 2014) We calculated pairwise F_ST_ s between demes using BEDASSLE (Bradburd et al. 2013) and, to test isolation by distance, performed a Mantel test using the R package adegenet (Jombart 2008).

### Estimated effective migration surface

We used Estimated Effective Migration Surface (EEMS; Petkova et al. 2015) to visualize population structure and spatially estimate areas of higher or lower than average gene flow across the landscape. EEMS models migration across the landscape by inferring migration rates among demes and represents these rates visually to offer insight into areas of low migration. We calculated the matrix of genetic dissimilarities between spatial locations with bed2diff pipeline. We used the Google Maps API v.3 tool (available at http://www.birdtheme.org/useful/v3tool.html) to draw a polygon encompassing our sampled localities. We performed eight independent chains of 10 million MCMC iterations with a burn-in of 200,000 MCMC iterations. Each independent chain was run with 400 demes and a thinning of 9999. Using the rEEMSplots tools, we did not find evidence for the lack of chain convergence (Fig. S1). Results over independent chains were summarized using the rEEMSplots pipeline.

### Generating spatial predictors of gene flow

We selected five landscape predictors that have been hypothesized to influence genetic variation in the Cerrado: (i) geographic distance; (ii) slope as a proxy of geomorphological compartmentalization; (iii) major rivers and their tributaries; (iv) vegetational shifts due to climatic oscillations over the last 21 kyr, and (v) environmental niche suitability over the last 21 kyr. The landscape layers were created using the following approaches: (i) the geographic distance raster depicted a homogeneous layer where all the pixels were equal to one. This layer represents the null model of isolation by geographic distance, where the landscape does not differ in its effect on individual dispersal. (ii) To create the slope layer, we first downloaded an elevational raster from the WorldClim database (Hijmans et al. 2005) and then created a slope raster by using the function *terrain* implemented in the R package *raster* (Hijmans and van Etten 2012). We hypothesized that higher values of slope represented regions of reduced gene flow. (iii) For rivers and their main tributaries, we downloaded hydrography shapefiles from HydroSHEDS (available at https://hydrosheds.org). Specifically, we sought to account for river heterogeneity by using a raster based on flow accumulation, which describe the amount of upstream area draining into a downstream cell. The hydrography raster comprised major rivers across the study area and their main tributaries. (iv) For vegetational shifts, we used the vegetation dynamics model over the last 30,000 years proposed by Costa et al. (2018). Using random forest classification of major South America biomes coupled with palaeomodeling to infer biome stability, they predicted how major vegetation types in South America changed every 1000 years (resulting in 21 climate layers—from 21 kyr to 1 kyr). Because *N. brasiliensis* occurs in a savanna-like vegetation, we classified open areas as enhancers of gene flow and the other vegetations representing areas of reduced gene flow. The last environmental predictor (i.e., environmental niche suitability over the last glacial maximum) is described in detail in the next section.

### Environmental niche modeling

We used environmental niche modeling (ENM) to predict areas of suitability for *N. brasilensis* (fifth landscape predictor). Occurrence data were comprised of localities with genomic data (9 localities) and additional record points without genomic information. For the latter, we obtained 30 independent geographic distribution records from the literature and the Herpetological Collection, University of Brasília (CHUNB). We used the R package *spThin* (Aiello-Lammens et al. 2015) to filter geographic occurrences at a geographic distance of 30 km to avoid sampling bias.

To create ENMs, we downloaded environmental predictors from the WorldClim database (available at http://www.wordclim.org) at a spatial resolution of 2.5 arc-minutes (Hijmans et al. 2005). They comprised 19 environmental variables that are related to patterns of precipitation and temperature. Next, we used the variance inflation factor to detect multicollinearity among environmental variables and kept only non-correlated variables. After this analysis the following variables were retained: mean diurnal range (BIO2), isothermality (BIO3), temperature seasonality (BIO4), mean temperature of wettest quarter (BIO8), mean temperature of warmest quarter (BIO10), precipitation of wettest month (BIO13), precipitation of driest month (BIO14), precipitation seasonality (BIO15), precipitation of warmest quarter (BIO18), precipitation of coldest quarter (BIO19).

ENMs were created using the maximum entropy algorithm MaxEnt (Phillips et al. 2006). To tune and evaluate ENMs models, we first selected 10,000 random background points and then chose one of the six feature classes combinations (L, H, LQ, LQH, LQHP, and LQHPT; L = linear, H = hinge; Q = quadratic, P = product; T = threshold) based on AIC values using the functions *randomPoints* and ENMevaluate, respectively, implemented in the R package *ENMeval* (Muscarella et al. 2014). The area under the curve was used to assess model performance. After constructing ENMs, we used the best fit model to project habitat suitability to seven time slices through the last 21 kyr: last glacial maximum (ca. 21 kyr), Heinrich Stadial 1 (17.0–14.7 kyr), Bølling-Allerød (14.7–12.9 kyr), Younger Dryas Stadial (12.9–11.7 kyr), early-Holocene, Greenlandian (11.7–8.326 kyr), mid-Holocene, Northgrippian (8.326–4.2 kyr), and late-Holocene, Meghalayan (4.2–0.3 kyr). All these historical environmental layers were downloaded from PaleoClim (available at http://www.paleoclim.org; Brown et al. 2018).

### Inferring landscape effects via optimized IBR models

We inferred the role of landscape features on gene flow using the recently developed R package *Radish* (Peterman and Pope 2021; https://github.com/nspope/radish). *Radish* approximates the likelihood of the genotype data conditional on an IBR model through regression of genetic distances onto resistance distances, with a correlation structure designed to account for the dyadic nature of pairwise measurements (Clarke et al. 2002). *Radish* optimizes resistance distance as a parameterized function of spatial covariates (in raster form): in particular, geographic distance, rivers, slope, habitat shifts, and environmental niche suitability. It then finds the maximum likelihood estimates of the weights associated with each spatial covariate, by profiling out nuisance parameters associated with the measurement model (i.e., the dyadic regression). We used our empirical dataset to calculate a genetic distance metric (F_ST_) among all demes using the R package *BEDASSLE* (Bradburd et al. 2013). Next, we used *Radish* to perform model selection and select the best model based on the lowest Akaike Information Criterion (AIC) for the same set of twelve models described above. MLPE was used rather than NMLPE because genetic dissimilarity was measured between populations, rather than between individuals nested within populations. For habitat shifts and environmental niche suitability, we used a single raster that represented the overall stability over the last 21 kyr. We used such a map because time slices do not represent independent hypotheses.

### Exploring machine learning in landscape genetics

Recently, Schrider and Kern (2018) promoted the incorporation of supervised machine learning (SML) techniques into evolutionary genetics. SML is a subfield of artificial intelligence concerned with training a predictive model from a pre-classified dataset (i.e., a dataset where the true label is known for all records). Similar to other spatial disciplines such as biogeography or phylogeography, landscape genetics is a historical discipline in the sense that the inferences that the researcher seeks to make are derived from analyses that cannot be experimentally replicated. Theory predicts that the interaction of molecular processes (e.g., Mendelian segregation, recombination, and point mutation) with demographic (e.g., population size change) and evolutionary (e.g., gene flow, selection) processes generate complex patterns of contemporary genetic polymorphism. Since these processes can be modeled effectively (Hudson 2002), simulation is a feasible means to circumvent the lack of experimental replication, allowing researchers to create realistically labeled datasets using robust and flexible simulation routines (e.g., Haller and Messer 2019; Landguth and Cushman 2010).

Simulation based approaches such as approximate Bayesian computation (ABC) have long been used in evolutionary genetics. The standard approach to ABC simulates a prior distribution under a specified model of demographic history, calculates summary statistics from each simulated dataset, and retains the small portion of the prior that closely match the summary statistics from the empirical data. This posterior distribution can be used to estimate parameters (e.g., Pritchard et al. 1999) or, if prior distributions are simulated under multiple demographic models, to calculate the posterior probability of a given model (e.g., Fagundes et al. 2007). Similarly, preclassified datasets can be created for SML using coalescent simulations under different landscape models. Rather than relying on statistics such as F_ST_ that summarize the data, an algorithm (e.g., random forest, support vector machine, artificial neural network) is used to train a predictive model by learning important features from the simulated datasets. Finally, the predictive model is used to calculate the relative probability of the set of the simulated models given the empirical dataset. SML has some potential advantages over ABC, for example it may be less prone to the curse of dimensionality (but see Pudlo et al. 2016) and may require fewer simulations because it does not include a rejection step (Schrider and Kern 2018).

Among the various SML analytical techniques, convolutional neural networks (CNNs) have been recently applied to several biological questions, ranging from detecting natural selection (Flagel et al. 2019; Torada et al. 2019) and reconstructing phylogenetic and phylogeographic history (Suvorov et al. 2020; Fonseca et al. 2021) to song annotation and individual recognition (Ferreira et al. 2020). CNNs are a class of artificial neural networks widely used to analyze visual images. Importantly, different from other approaches, CNNs allow the inference of what landscape features have driven genetic differentiation directly from the DNA alignment, containing all the genetic variation from sampled individuals across the study area. CNNs eliminate the necessity of calculating genetic summary statistics, as demonstrated by Flagel et al. (2019). Therefore, CNNs enable alternative processes that potentially influence contemporary genetic patterns to be directly compared.

### Landscape model selection using convolutional neural network (CNN)

We used a CNN to calculate the relative probability of twelve spatially explicit models given the empirical dataset. Each model corresponded to a unique combination of landscape predictors (Table 1): *isolation by distance*: model 1 – geographic distance; *isolation by resistance*: model 2 – slope; model 3 – rivers; model 4 – habitat shifts; model 5 – environmental niche suitability; model 6 – slope and rivers; model 7 – slope and habitat shifts; model 8 – slope and environmental niche suitability; model 9 – rivers and habitat shifts; model 10 – rivers and environmental niche suitability; model 11 – slope, rivers, and habitat shifts; model 12 – slope, rivers, and environmental niche suitability. We did not include habitat shifts and environmental niche suitability in the same model because they were built using the same set of environmental predictors, making them non-independent hypotheses. Because IBD is a special case of IBR, geographic distance is implicitly incorporated in IBR models.

**Table 1:** Results from AMOVA showing the variance within and between demes and the population structure recovered by sNMF.

Source	df	Source of variaton	Sum of squares	Mean square	% variation explained
Demes	8	Within demes	29,625.79	3703.22	35.1
	43	Between demes	15,038.02	349.72	64.9%
	51	Total	44,663.8	875.76	100%
sNMF	4	Within demes	26,088.98	6522.21	38.4%
	47	Between demes	18,574.93	395.21	61.6%
	51	Total	44,663.81	875.76	100%

We used Fastsimcoal2 (Excoffier et al. 2013) to simulate datasets for each model. We created customized models that mirrored our empirical dataset regarding the number of SNPs, localities, and individuals per locality. We simulated a total of 2500 data examples under each model. Simulations were performed under an island model in which an ancestral population split into 39 demes 21,000 years ago, representing the total number of localities and the oldest landscape layer, respectively. We sampled a value for ancestral population size from a uniform distribution of 20,000–50,000 haploid individuals for each simulation. Population sizes of each individual deme were sampled from a uniform distribution with minimum and maximum values set to 5 and 100, respectively. We simulated a total of 4364 SNPs per individual, which is the number of SNPs per individual in the empirical dataset.

For each simulated data example, a migration matrix representing the expected amount of the gene flow was calculated for each landscape hypothesis. The simulations included localities with and without genetic data, with the latter included to account for their impact on the genetic variation in sampled localities and to create a more continuous migration model. However, during the simulation procedure, we only sampled SNPs from localities with genomic data. To create the migration matrix, we first sampled a value of landscape effect for each landscape feature of a given model, ranging from 2 to 5, using a uniform distribution. Based on preliminary runs, these values represent low to high landscape effect. Next, we multiplied each pixel in the landscape raster by the landscape effect. If a model included two or more layers, we summed them to create a unique landscape layer, as recently recommended by Peterman and Pope (2021). We used this composite layer to calculate the least-cost path (i.e., resistance distance) among all points using the *costDistance* function implemented in the R package *gdistance* (van Etten 2017). Next, we converted the resultant resistance matrix to a migration matrix using the equation (1/x)^3^, where *x* is the resistance distance between two geographic localities. We used such an equation because we expected that migration among demes decreases exponentially as resistance distance increases due to the limited dispersal capacity of lizards. These transformations were selected because they were necessary to produce simulated datasets that matched the empirical data in aspects such as the number of SNPs and population genetic structure. Our assumption throughout was to utilize the information that we could quantify (e.g., landscape resistance, genetic variation, population structure) to simulate data that matched the observed data as closely as possible. Simulations were compared to the observe data using principal components analysis (PCA). In preliminary runs, we raised the resistance matrix to a second power, however, many simulated datasets had no genetic structure, likely due to the high migration rate. Lastly, because there is no available information about the dispersal ability of the focal species in the landscape, we created a parameter to account for this uncertainty. We sampled this parameter from a uniform distribution (minimum: 0.1 and maximum: 0.3) and used it to multiply the migration matrix. In models containing the vegetation shifts or environmental niche suitability hypothesis, we repeated this step for each of the layers and updated each migration matrix based on their historical period in the simulations.

Finally, we converted the genetic alignment of each simulated dataset into a biallelic matrix, with rows and columns representing individuals and individual SNPs, respectively. The major allele was labeled as “0” and the minor allele as “1” and then, this matrix was converted into a black and white image with each pixel corresponding to a SNP. Finally, columns (representing SNPs) were sorted from higher to lower allele frequency and rows (representing individuals) were organized from deme 1 to deme 9 (numbers in Fig. 1).

**Fig. 2:**
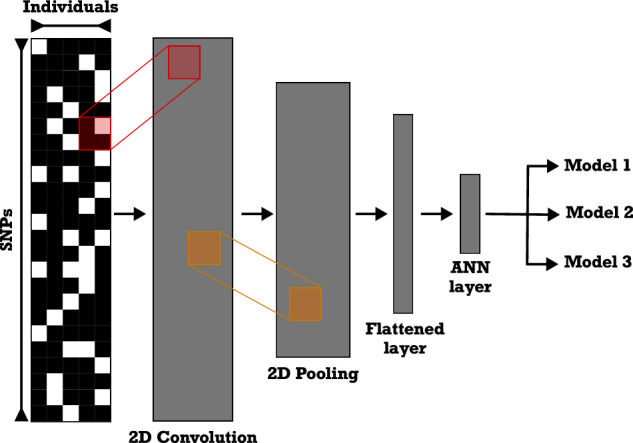
Illustration of the architecture used in to build the CNN for the analysis of simulated landscape data. From left to right, the figure depicts the convolution, pooling, and flattening steps that are used to transform the data into layers that can be analyzed via the artificial neural network.

We built all CNNs with the Keras python library (https://keras.io) using the following two-dimension architecture (Fig. 2): convolution layer (kernel = 3 × 1), a two-dimensional maximum pooling layer (kernel = 3 × 1), a two-dimensional convolution layer (kernel = 3 × 1), and a two-dimensional maximum pooling layer (kernel = 3 × 1). Then, the output layer from the last pooling was flattened and fully connected to a layer with 100 neurons, followed by another with 40 neurons, and an output layer with twelve neurons – each neuron on the last layer corresponded to a different model. We used the rectified linear unit activation function (ReLU) for all layers, except for the last one in which we implemented a softmax function. The softmax function is a generalization of the logistic function useful for multiclass prediction. CNN was compiled using the Adam optimization procedure, a categorical cross-entropy loss function, and a mini-batch size of 100 and then run for ten epochs. We used 80% (24,000 data examples; 2000 data examples per model) of the simulated datasets for training the model and the remaining 20% (6000 data examples; 500 data examples per model) to evaluate model accuracy. Finally, the trained model was used to predict the empirical dataset. For a more detailed information on CNNs and deep learning, we recommend Lecun, et al. (2015) and Flagel et al. (2019).

To evaluate model accuracy under each model, we created a confusion matrix and calculated precision [TP/(TP + FP); where TP = true positive and FP = false positive] and recall [TP/(TP + FN); where TP = true positive and FN = false negative] values. We evaluated the calibration of the softmax function by computing the absolute output probability of each simulation on each model on the test dataset and assigned each value into five classes (0%–20%, 20%–40%, 40%–60%, 60%–80%, 80%–100%) because a well-calibrated model should have the probability associated with the predicted label proportional to the training dataset (Guo et al. 2017). Finally, we simulated an additional 2500 data examples under the best model and calculated F_ST_ among all localities. Then we used a PCA to summarize the genetic variation in the simulated datasets to ensure that each model produced a range of genetic data that contained the variation observed in the empirical dataset.

**Fig. 3:**
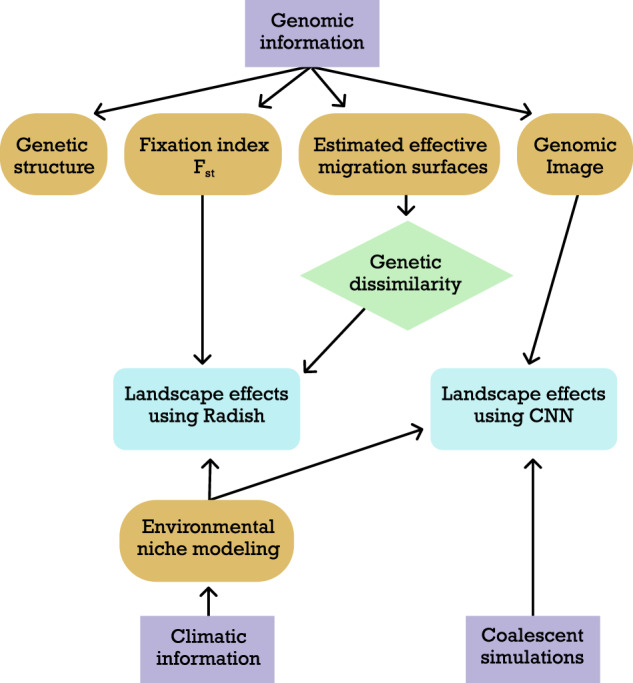
Analytical workflow diagram. this schematic outlines the sequential steps and data progression used in our study, with directional arrows illustrating the flow and transformation of data at each stage of the analysis.

The analyses described in the above paragraphs are intended to be complementary, and we anticipate that inferences about the focal system will be improved by interpreting the results from a given analysis in the context provided by other results. For example, results from the environmental modeling will inform the analyses that have been designed to detect landscape effects, as will F_ST_ calculations and genetic dissimilarity (Fig. 3).

### Assessing potential bias on study design

Because one potential limitation in our study was that most of our samples came from the western distribution of *N. brasiliensis*, we evaluated how the number of demes and sequences influence the performance of the CNN. To assess how this bias affected our predictive model, we re-simulated datasets for each of the twelve landscape models. Simulations were performed using the same priors and conditions as described above. We built simulated datasets to assess the effect of (i) genetic and (ii) geographic sampling and their interaction. First, we used the same number of demes with genetic information (i.e., nine localities) as observed in the empirical dataset and applied two genetic sampling strategies: 2 and 20 sequences within each deme, representing 1 and 10 individuals, respectively. Next, instead of sampling genetic information for only nine demes, we gathered genetic information for all the 39 demes under the same two genetic sampling strategies (i.e., 1 and 10 individuals). We assessed model accuracy under each model on each scheme using a combination of confusion matrix, precision, recall, and model calibration. Importantly, we compared the accuracy of these datasets with the simulated dataset that mirrored our empirical data example (i.e., 9 localities and an unbalanced genetic sampling). This experiment represents a gradient of a highly desirable dataset (39 demes and 20 individuals/deme) to lesser desirable datasets (39 demes and 1 individual/deme; 9 demes and 20 individuals/deme) to a very problematic dataset (9 localities and 1 individual/deme).

## Results

### Genetic diversity and differentiation

Our final dataset contained a total of 4365 unlinked SNPs. The overall values for expected (H_e_) and observed heterozygosity (H_o_) were 0.09624 ± 0.145 and 0.07056 ± 0.05, respectively. Genetic distance (F_ST_) among localities varied from 0.08 to 0.75 (0.5 ± 0.14). Plots of the genetic distance against each landscape feature are shown in Fig. S2. AMOVA showed that there is more genetic variance between demes and the populations recovered by sNMF (Table 1; 64.9% and 61.6%, respectively) than within these populations (Table 1; 35.1% and 38.4%, respectively). Pairwise F_ST_s as calculated by BEDASSLE are shown in Table 2.

**Table 2:** Table reporting fixation indexes (F_ST_) calculated using BEDASSLE for nine demes.

	1	2	3	4	5	6	7	8	9
1	0	0.7807083	0.3579397	0.65438131	0.7816377	0.63948448	0.4810129	0.6354899	0.585507
2	0.7807083	0	0.3934924	0.67746453	0.4527906	0.65402307	0.5425239	0.7100498	0.6069379
3	0.3579397	0.3934924	0	0.30666951	0.2904241	0.33963419	0.4002934	0.4470513	0.4884074
4	0.6543813	0.6774645	0.3066695	0	0.631679	0.07997084	0.5178014	0.6098282	0.5744222
5	0.7816377	0.4527906	0.2904241	0.63167898	0	0.6147726	0.4702399	0.6343171	0.5704431
6	0.6394845	0.6540231	0.3396342	0.07997084	0.6147726	0	0.525684	0.6023262	0.5724203
7	0.4810129	0.5425239	0.4002934	0.51780138	0.4702399	0.52568397	0	0.3480316	0.4762887
8	0.6354899	0.7100498	0.4470513	0.60982823	0.6343171	0.60232615	0.3480316	0	0.5265446
9	0.585507	0.6069379	0.4884074	0.57442222	0.5704431	0.57242026	0.4762887	0.5265446	0

The numbers correspond to deme localities shown in Fig. 1.

### Genetic structure

**Fig. 4:**
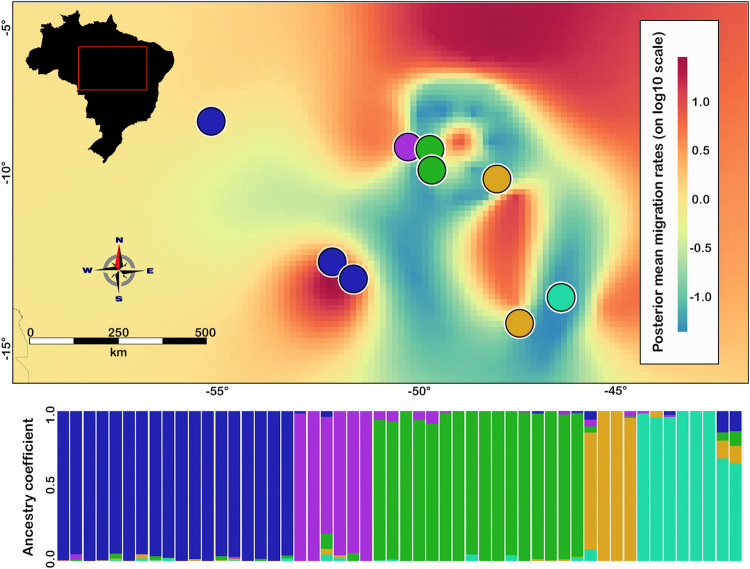
Map showing the result of the sNMF clustering analysis (colored circles) and the effective migration rates estimated using EEMS (background color). Barplot represents the ancestry coefficient recovered in sNMF. In the background, migrates rates varies from lower (blue) to higher (red). EEMS represent the mean migration rate across 8 independent runs.

**Fig. 5:**
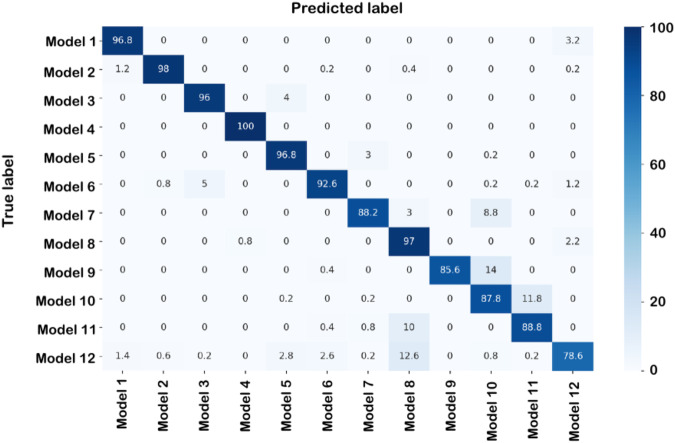
Confusion matrix measuring the accuracy of the trained predictive model. Numbers represent percentages, which were calculated based on 500 images for each model. Overall accuracy = 89.5%.

The Mantel test showed that geographic distance is correlated with genetic (*P* < 0.01; 999 permutations). The sNMF clustering analysis supported five geographically structured populations (Fig. 2). The plotted cross-entropy values indicated a valley at *K* = 5, with lesser and greater values of *K* showing higher cross-entropy values (Fig. S3). The bar plot depicting the ancestry coefficient for *K* = 5 indicated some admixture among populations once the ancestry coefficient is shared between different populations (Figs. 4 and 5). The cyan population is distributed in an enclave of Seasonally Dry Tropical Forest. Green and purple populations are found in low landscape in the Cerrado. Finally, we found two populations that are more widespread in the landscape (blue and orange populations; Fig. 4).

The migration surface estimated by EEMS showed evidence of several regions of lower gene flow than expected by IBD (Fig. 4). Overall, this surface supported the genetic structure recovered by sNMF. For example, although localities from the orange populations are far apart, they are linked via a higher migration rate. In contrast, the cyan population is disconnected from one of the orange populations by a region of lower migration, albeit being geographically close. Both sNMF and EEMS did not show any visual concordance between population structure and physical barriers.

### Optimized IBR models

The maximum likelihood of the genotypic data conditional on the IBR models was calculated using *Radish*. After calculating AIC, model selection was conducted using optimized IBR models in the set of twelve landscape scenarios (models 1–12). This procedure recovered model 1 as the best model with the lowest AIC value (AIC = −113.85; Table 3). Model 1 represented our null model of isolation by distance (i.e., no effect of landscape on dispersal). Although other models had ΔAIC scores lower than two (models 2, 3, and 5), they did not result in a substantial improvement in fit relative to the null model (*p* > 0.05 using likelihood ratio tests).

**Table 3:** Model probability values of the trained convolutional neural network (CNN) and AIC values for the optimized IBR models.

Model	Landscape features	CNN	Optimized IBR models		
		Probability	AIC	ΔAIC	*w*_i_
Model 1	Geographic distance	**1**	−**113.9**	**0**	**0.252**
Model 2	Slope	0	−111.9	1.92	0.097
Model 3	Rivers	0	−112.1	1.71	0.107
Model 4	Habitat shifts	0	−111.9	2	0.093
Model 5	Environmental niche suitability	0	−112.5	1.39	0.126
Model 6	Slope + Rivers	0	−110.4	3.44	0.045
Model 7	Slope + Habitat shifts	0	−109.9	3.92	0.036
Model 8	Slope + Environmental niche suitability	0	−111.7	2.16	0.086
Model 9	Rivers + Habitat shifts	0	−110.2	3.66	0.040
Model 10	Rivers + Environmental niche suitability	0	−110.9	2.94	0.058
Model 11	Slope + Rivers + Habitat shifts	0	−108.4	5.42	0.017
Model 12	Slope + Rivers + Environmental niche suitability	0	−110.3	3.55	0.043

The overall accuracy of the CNN model was 89.5%, The best model in each approach is highlight in bold.

### Convolutional neural network

The simulation trained CNN recovered isolation by distance (model 1) as the best fit model given the empirical dataset with 100% probability (Table 3). All other scenarios had a 0% probability according to our predictive model. Our predictive model reached high values of precision and recall (Table S1). The trained CNN model had a high accuracy when predicting the test dataset, with an overall accuracy of 89.5% (Fig. 5). Simulated datasets under the best model produced summary statistics consistent with our empirical dataset (e.g., Fig. S4). Also, the calibration analysis showed that the predictive model is well-calibrated and that individual probabilities are proportional to training dataset accuracy (Fig. S5).

### Assessing potential bias on study design

Because most of our samples were from the western Cerrado, we built four additional CNNs models to assess the effect of the number of demes and individuals within each deme, reflecting potential bias on real datasets. Both the number of demes and the number of sampled individuals within each deme play a role in model accuracy. Simulating nine demes and sampling two sequences from each deme resulted in poor model performance in terms of overall accuracy (77.3%; Fig. S6) and precision and recall (Table S1). Increasing the sampling strategy to 10 individuals within deme while keeping the same nine demes drastically increased the accuracy of the model. (91.1%; Fig. S7, Table S1). For 39 demes, both sampling strategies, two sequences or 20 sequences per deme (i.e., 1 or 10 individuals), recovered satisfactory values of model performance (92% and 97.8%, respectively; Figs. S8–S9, Table S1). The calibration of each predictive model is presented in Figs. S10–S13 and showed that only the model with nine demes and two sequences per deme performed poorly. Based on these results, we conclude that our sampling scheme is adequate to capture landscape variation and produce a reliable inference, although we acknowledge that additional sampling is likely to improve the analysis.

## Discussion

Simulation-based methods in evolutionary genetics attempt to infer how historical processes acting across the landscape have influenced extant genetic diversity (Knowles and Alvarado-Serrano 2010; Pelletier and Carstens 2016). While our investigation is limited to some extent by its sampling, which potentially explains curious results such as the higher F_ST_ values, the study design facilitates the computationally complex analyses such as the CNN used here. After reviewing briefly reviewing the history of the region occupied by the focal species, we explore the potential application of machine learning methods to landscape genetic investigations.

### Landscape effects on genetic structure and gene flow in *Norops brasiliensis*

In any investigation into an empirical system, inferences are a product of interpretation of the result given what is known about the history of the region that the focal species occupies. The Cerrado contains many features that have been implicated in other investigations as important factors that influence intraspecific genetic diversity. For example, several investigations have demonstrated that river systems influence genetic variation (Funk et al. 2007; Bartáková et al. 2015; Lanna et al. 2020), whether by acting as allopatric barriers (Nazareno et al. 2017; Naka and Brumfield 2018) or by facilitating gene flow (Thom et al. 2020; Fonseca et al. 2021). Similarly, the topography of the Central Brazil plateau, which was largely caused by erosion during the Neogene, compartmentalized the Cerrado landscape and created younger valleys characterized by more heterogeneous forest assemblages between older plateaus, harboring savanna-like vegetation (Colli 2005; Werneck 2011). The varied topography has been implemented as a cause of population genetic structure in other species (Camurugi et al. 2021; Domingos et al. 2014; Giugliano et al. 2013; Oliveira et al. 2018; Prado et al. 2012). Finally, Pleistocene climate oscillations are a prominent driver of intraspecific diversification in the Neotropics (Carnaval et al. 2009; Gehara et al. 2017) and have been identified as a driver of genetic structure within other species (Vasconcellos et al. 2019; Camurugi et al. 2021).

Our motivation for designing our SML approach to data analysis was due in large part to our desire to infer the relative influence of these features on genetic diversity in *N. brasiliensis*. However, few of these features had a demonstrable effect in the SML results, for reasons that may be related to how they were incorporated into our models. Rivers were not recovered as an important landscape feature that population genomics of *N. brasiliensis*. While rivers are highly heterogeneous systems in evolutionary time, our models assumed that they remained unchanged over the last few thousands of years. We believe this is a reasonable assumption for the Central Brazil plateau river drainages since large-scale river rearrangement can take many thousands to millions of years to occur (Mabesoone, 1994; Hoorn et al. 2010), but it does represent a potential shortcoming of our model. We also did not identify habitat shifts or niche suitability as factors that exerted a large effect on genetic diversity in *N. brasiliensis*. Previously, Fonseca et al. (2021) showed that conspicuous effective population size expansions in *N. brasiliensis* occurred and hypothesized that these were responses to Pleistocene climatic oscillations. Since ecological niche models assume that species’ environmental preferences are conserved through time and usually do not account for adaptive processes, this result would either imply that demographic size changes were not a response to these oscillations or that the environmental preferences in *N. brasiliensis* have changed. It is possible that local adaptation and/or phenotypic plasticity could potentially maintain a more stable range under a less favorable climate, but these were also factors that were not incorporated into our models.

Our population assignment analysis found evidence for five geographically distributed populations (Figs. 2 and S3). Results from the AMOVA indicate that most of the genetic variance is partitioned among populations, while the EEMS analysis revealed apparent regions of low gene flow. Furthermore, the population structure observed in *N. brasiliensis* is similar to that found in other species in the Cerrado (Prado et al. 2012; Santos et al. 2014; Guarnizo et al. 2016). Taken in total, these results support an inference that the varied topography of the Cerrado leads to population genetic structure in the focal species. However, we did not find evidence that *landscape topography* was the aspect of the landscape that led to the genetic structure, as results from the optimized IBR models and SNL analysis each demonstrated that genetic structure in *N. brasiliensis* can be explained largely by geographic distance. While our investigation, like that of. Camurugi et al. (2021), used a spatial analysis to identify whether slope has influenced patterns of gene flow, it may be that slope is not an effective proxy of landscape topography. It remains the case that some features of the landscape are difficult to model in a manner that correspond to the particular life history characteristics of the focal species.

Perhaps it shouldn’t be surprising that genetic variation in the focal taxon is largely explained by geographic isolation. Isolation by distance is an important phenomenon that has long been recognized as a key influence on genetic variation (Wright 1943). Recent global surveys have demonstrated that IBD is ubiquitous in its influence across the tree of life (Sexton et al. 2014; Pelletier and Carstens 2018). The fact that the Cerrado covers a large area in the Central Brazilian Plateau, in combination with the limited dispersal capability of lizards, can likely explain the prevalence of IBD in *N. brasiliensis*. It is possible that some combination of the strong signal of IBD and the less than comprehensive sampling made it difficult to detect any effect of slope, rivers, habitat shifts or, environmental niche suitability in the genetic variation of *N. brasiliensis*.

### Model selection in *N. brasiliensis*

**Fig. 6:**
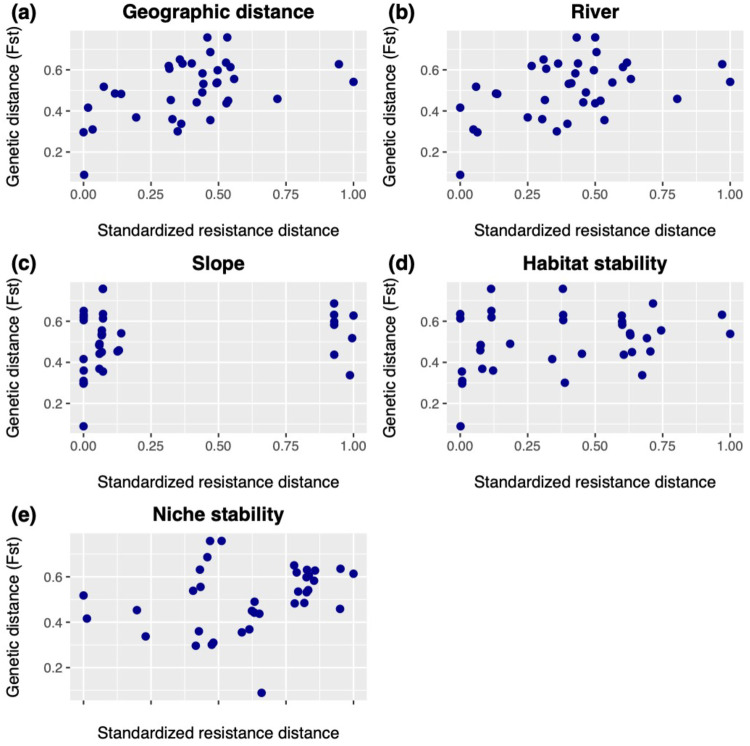
Plots showing relationship between genetic distance and landscape distances. Comparison of genetic distance, measured by estimates of F_ST_ among demes, and five landscape features: (**a**) geographic distance, (**b**) rivers, (**c**) slope, (**d**) habitat stability, (**e**) niche stability.

We evaluated models that incorporated features of the landscape that may influence genetic diversity in our focal species so that we could identify the feature(s) that exerted the greatest influence following Anderson (2008). This approach, which is widely used in phylogeographic research (Fagundes et al. 2007; Satler and Carstens 2016; Smith et al. 2017), uses statistical model selection to identify the model that has the highest probability given the data. In our case, the IBD model (i.e., model 1) was found to have the highest probability by both the IBR and the SML analyses. Importantly, model selection can quantify the support for all models given the data. For example, the results of the IBR analysis indicate that model probabilities (*w*_i_) are spread across multiple models to the extent that there isn’t a single landscape feature that can account for the observed genet\ic data (Table 3). In contrast, the SLM approach finds that the model which corresponds to isolation by distance contains all the model probability (Table 3). We are uncertain as to which of these results we should favored. While it seems intuitive that many features of the landscape should influence genetic variation within *N. brasiliensis* (e.g., Fig. 6), it may be that these features largely covary with geographic separation and that this covariance does not influence the SML approach. By conducting landscape analyses in a model selection framework we avoid subjective interpretations of the results and, consequently, makes them less prone to overinterpretation (Knowles and Maddison 2002) and confirmation bias (Nickerson 1998).

### Machine learning methods in landscape genetics

Landscape genetics has expanded its analytical toolkit to take advantage of recent advances in genomic sequencing (e.g., Bradburd et al. 2018; Petkova et al. 2015). However, researchers have not fully explored all potential analytical tools to tackle the challenges posed by the massive size of genomic datasets. Simulations coupled with machine learning have the potential to incorporate salient features of the landscape into analytical frameworks, allowing the simulation of customized datasets under different hypotheses that can resemble the details of any empirical system, including the number of demes (sampled and non-sampled), number of individuals per deme, and number of SNPs. Models that represent hypotheses that are tailored to the focal system can be implemented and tested to ensure their suitability and sensitivity (Carstens et al. 2022). In this investigation, we demonstrate that CNN can be an efficient and accurate tool for use in exploring potential landscape effects on genetic variation.

One appealing aspect of the approach used here is how the genomic data are summarized. The CNN uses a series of transformations to convert an alignment of DNA sequence data into an image that captures salient features of the genomic variation (Flagel et al. 2019). In contrast to allele frequency spectra (e.g., Gutenkunst et al. 2009) or summary statistics, representing the data as an image allows the researcher to borrow the suite of computational tools that has been developed for image processing, leading to an efficient evaluation of the genetic data. Of course information can be lost when genetic data is reduced to any summary statistic, even when dozens of summary statistics are used in landscape genetics (Shirk et al. 2017). This loss of information can complicate inference. For example, many investigations have compared genetic distance metrics, such as Wright’s *F-*statistics, to landscape features (Fig. 6). A casual interpretation of this figure might suggest that each of these factors has a comparable influence on genetic diversity within *N. brasiliensis*. There is no perfect summary of genetic data, as each statistic has inherent advantages and disadvantages. In practice, researchers too often rely on historical inertia or arbitrary choices of summary statistics for their investigation, although newer methods address this by directly modeling allele frequencies (e.g., Vanhove and Launey 2023). Machine learning approaches can thus provide a useful complement to the use of statistics to summarize genetic variation.

One disadvantage of the CNN approach is that it can be computationally demanding. For reference, it took from 30 min to 7 h (models with habitat shifts and environmental niche suitability were more computationally demanding because of the high number of layers) to simulate 2500 images under each model in a supercomputer using 40 cores in parallel. In contrast, the optimized IBR models implemented in Radish took less than 15 min to fit all the models on a Mac mini, 1.6 GHz Intel Core i5, 8 GB RAM. Importantly, once a CNN is trained it takes little additional effort to explore questions related to sampling. Here we explored the potential effects of limited sample sizes, a feature that is an unfortunate reality for many empirical datasets due to the high cost of collecting samples for widely distributed species across complex landscapes. Even though our sampling was biased towards the western distribution range of *N. brasiliensis*, the results of our analysis assessing the potential bias in study design indicated that our empirical samples encompassed a sufficient degree of landscape variation to fit a predictive model.

While machine learning has been applied to a range of questions (Flagel et al. 2019; Smith and Carstens 2020; Suvorov et al. 2020; Fonseca et al. 2021), these approaches have tremendous potential for landscape genetics. For example, Burbrink et al., (2021) used an artificial neural network to infer how landscape and environmental features predicted the genetic structure of North American rat snakes (*Pantherophis obsoletus* complex). They showed that their predictive model was highly accurate in predicting genetic distance (accuracy was greater than 90%). A complementary approach is implemented in the R package *ResistanceGA* (Peterman 2018), which uses a genetic algorithm to optimize resistance surfaces based on pairwise genetic data and resistance distances. Pless et al. (2021) implemented a random forest classified to map landscape connectivity in the invasive mosquito *Aedes aegypti* (vector of several diseases, including dengue and Zika) in North America. Kittlein et al. (2021) provided another example when they trained a CNN to predict local F_ST_ and mean allelic richness. Thom et al. (2021) used a bidimensional stepping-stone model with artificial neural network to show that populations in tropical mountains in the Brazilian Atlantic Forest have higher rates of gene flow. In related disciplines, ABC-RF is now routinely being used in historical demographic studies (Pudlo et al. 2016; Smith et al. 2017; Smith and Carstens 2020).

## Conclusions

Our study showed that geographic distance is an important predictor of genetic structure in *N. brasiliensis*. Using a novel CNN-based approach, we could not detect the effects of slope, rivers, habitat shifts, and environmental niche suitability on genetic differentiation. Other results suggest that some of these features may be important and highlight the need for additional exploration of the most effective ways to incorporate machine learning methods into landscape genetics.

### Supplementary information

Supplemental materials

## Data Availability

All data and scripts are openly available at GitHub (https://github.com/emanuelmfonseca/landscape-effects-neotropical-lizard).
