# Supplementary material for: Genetic structure and landscape effects on gene flow in the Neotropical lizard Norops brasiliensis (Squamata: Dactyloidae)
Source: Heredity (Edinb). 2024 Apr 4;132(6):284–95. doi: 10.1038/s41437-024-00682-5 (PMC11166928; doi:10.1038/s41437-024-00682-5)
Supplement: Supplementary file 1 — Supplemental materials [file 41437_2024_682_MOESM1_ESM.docx]

###

Supporting information

**Genetic structure and landscape effects on gene flow in the Neotropical lizard *Norops brasiliensis* (Squamata: Dactyloidae)**

Emanuel M. Fonseca^*^, Nathaniel S. Pope, William E. Peterman, Fernanda P. Werneck, Guarino R. Colli, Bryan C. Carstens

*Corresponding author: emanuelmfonseca@gmail.com; Department of Evolution, Ecology and Organismal Biology, The Ohio State University, 318 W. 12th Ave, Columbus, OH 43210

###

**Table S1.** Precision and recall values of the trained convolutional neural networks (CNN) used to test the influence of the study design. The overall accuracies of the predictive models were 77%, 94.6%, 94.9%, 98.9%, respectively.

| **Model** | **Landscape features** | **9 demes** | |  | **39 demes** | |
| --- | --- | --- | --- | --- | --- | --- |
|  |  | **2 sequences** | **20 sequences** |  | **2 sequences** | **20 sequences** |
|  |  | **Precision/Recall** | **Precision/Recall** |  | **Precision/Recall** | **Precision/Recall** |
| Model 1 | Geographic distance | 0.94/0.91 | 1.00/0.98 |  | 0.97/0.90 | 1.00/0.99 |
| Model 2 | Slope | 0.67/0.50 | 0.86/0.93 |  | 0.89/0.98 | 0.98/0.99 |
| Model 3 | Rivers | 0.77/0.76 | 0.94/0.85 |  | 0.96/0.85 | 1.00/0.98 |
| Model 4 | Vegetation shifts | 0.58/0.70 | 0.91/0.81 |  | 0.97/0.79 | 0.99/0.96 |
| Model 5 | Climatic suitability | 0.81/0.91 | 0.96/1.00 |  | 0.89/0.98 | 0.99/1.00 |
| Model 6 | Slope + Rivers | 0.73/0.81 | 0.98/1.00 |  | 1.00/1.00 | 1.00/1.00 |
| Model 7 | Slope + Vegetation shifts | 1.00/0.99 | 1.00/1.00 |  | 1.00/0.99 | 1.00/0.99 |
| Model 8 | Rivers + Vegetation shifts | 0.86/0.84 | 0.98/0.98 |  | 0.98/0.96 | 0.99/0.99 |
| Model 9 | Slope + Rivers + Vegetation shifts | 0.65/0.72 | 0.97/0.98 |  | 0.92/0.99 | 0.98/1.00 |
| Model 10 | Slope + Climatic suitability | 0.69/0.93 | 0.91/0.99 |  | 0.94/0.99 | 0.97/1.00 |
| Model 11 | Rivers + Climatic suitability | 0.76/0.68 | 0.96/0.96 |  | 0.95/0.97 | 0.99/0.98 |
| Model 11 | Slope + Rivers + Climatic suitability | 0.93/0.50 | 0.92/0.94 |  | 0.92/0.96 | 0.99/0.98 |

**
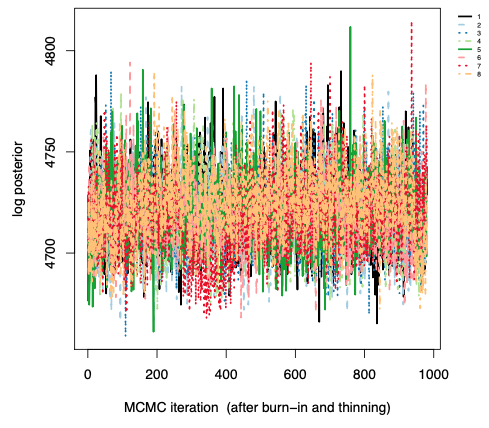
Figure S1.** Plot showing any evidence for the lack of convergence across eight independent runs in EEMS.

**Figure S2.** The relationship between genetic differentiation and (a) geographic distance, (b) rivers, (c) slope, and (d) climate.

**Figure S3.** Plot showing cross-entropy values across differennt values of *K* in sNMF analysis.

**Figure S4.** Principal Components Analysis plots (PC1 X PC2) derived from the simulated distributions of summary statistics (F_ST_) from model 1 and observed dataset

**Figure S5**. Heatmap describing the output probability percentage under five probability classes for the CNN built using 9 localities (0%–20%, 20%–40%, 40%–60%, 60%–80%, 80%–100%). Numbers represent percentages, which were calculated based on 500 images for each model from the test dataset.

**Figure S6.** Confusion matrix measuring the accuracy of the trained predictive model using 9 demes and sampling 2 sequences per deme. Numbers represent percentages, which were calculated based on 500 images for each model. Overall accuracy = 77%.

**Figure S7.** Confusion matrix measuring the accuracy of the trained predictive model using 9 demes and sampling 20 sequences per deme. Numbers represent percentages, which were calculated based on 500 images for each model. Overall accuracy = 94.6%.

**Figure S8.** Confusion matrix measuring the accuracy of the trained predictive model using all 39 demes and sampling 2 sequences per deme. Numbers represent percentages, which were calculated based on 500 images for each model. Overall accuracy = 94.9%%.

**Figure S9.** Confusion matrix measuring the accuracy of the trained predictive model using all 39 demes and sampling 20 sequences per deme. Numbers represent percentages, which were calculated based on 500 images for each model. Overall accuracy = 98.9%.

**Figure S10**. Heatmap describing the output probability percentage under five probability classes (0%–20%, 20%–40%, 40%–60%, 60%–80%, 80%–100%) for the CNN built using 9 demes and sampling 2 sequences per deme. Numbers represent percentages, which were calculated based on 500 images for each model from the test dataset.

**Figure S11**. Heatmap describing the output probability percentage under five probability classes (0%–20%, 20%–40%, 40%–60%, 60%–80%, 80%–100%) for the CNN built using 9 demes and sampling 20 sequences per deme. Numbers represent percentages, which were calculated based on 500 images for each model from the test dataset.

**Figure S12**. Heatmap describing the output probability percentage under five probability classes (0%–20%, 20%–40%, 40%–60%, 60%–80%, 80%–100%) for the CNN built using 39 demes and sampling 2 sequences per deme. Numbers represent percentages, which were calculated based on 500 images for each model from the test dataset.

**Figure S13**. Heatmap describing the output probability percentage under five probability classes (0%–20%, 20%–40%, 40%–60%, 60%–80%, 80%–100%) for the CNN built using 39 demes and sampling 20 sequences per deme. Numbers represent percentages, which were calculated based on 500 images for each model from the test dataset.
